# Player position in American football influences the magnitude of mechanical strains produced in the location of chronic traumatic encephalopathy pathology: A computational modelling study

**DOI:** 10.1016/j.jbiomech.2021.110256

**Published:** 2021-01-23

**Authors:** K.A. Zimmerman, J. Kim, C. Karton, L. Lochhead, D.J. Sharp, T. Hoshizaki, M. Ghajari

**Affiliations:** a Computational, Cognitive and Clinical Neuroimaging Laboratory, Department of Brain Sciences, Hammersmith Hospital, Imperial College London, London, UK; b HEAD Lab, Dyson School of Design Engineering, Imperial College London, UK; c Neurotrauma Impact Science Laboratory, University of Ottawa, Canada; d Care Research & Technology Centre, UK Dementia Research Institute, London, UK

**Keywords:** Brain injuries, Traumatic, Brain concussion, Neuroanatomy, Tauopathies, Biomechanics

## Abstract

American football players are frequently exposed to head impacts, which can cause concussions and may lead to neurodegenerative diseases such as chronic traumatic encephalopathy (CTE). Player position appears to influence the risk of concussion but there is limited work on its effect on the risk of CTE. Computational modelling has shown that large brain deformations during head impacts co-localise with CTE pathology in sulci. Here we test whether player position has an effect on brain deformation within the sulci, a possible biomechanical trigger for CTE. We physically reconstructed 148 head impact events from video footage of American Football games. Players were separated into 3 different position profiles based on the magnitude and frequency of impacts. A detailed finite element model of TBI was then used to predict Green-Lagrange strain and strain rate across the brain and in sulci. Using a one-way ANOVA, we found that in positions where players were exposed to large magnitude and low frequency impacts (e.g. defensive back and wide receiver), strain and strain rate across the brain and in sulci were highest. We also found that rotational head motion is a key determinant in producing large strains and strain rates in the sulci. Our results suggest that player position has a significant effect on impact kinematics, influencing the magnitude of deformations within sulci, which spatially corresponds to where CTE pathology is observed. This work can inform future studies investigating different player-position risks for concussion and CTE and guide design of prevention systems.

## 1 Introduction

American football players are frequently exposed to head impacts, the accumulations of which is proposed to increase the risk of developing Chronic Traumatic Encephalopathy (CTE), a neurodegenerative disease (McKee et al., 2013; Mez et al., 2017). CTE is characterised by the accumulation of abnormal tau proteins along small blood vessels and in the depths of the sulci (McKee et al., 2016). Computational modelling of traumatic brain injury (TBI) allows us to predict patterns of brain deformation during the short time of impact (Ghajari et al., 2017; Kleiven and Hardy, 2002; Zhang et al., 2001). The distribution and magnitude of mechanical strain and strain rate have been informative in predicting post-traumatic pathology such as axonal injury and permanent tissue damage (Bain and Meaney, 2000; Morrison et al., 2003; Tagge et al., 2018), and they are likely to be informative in predicting the onset of CTE.

Recent studies show that computational modelling may have uses beyond predicting primary injury pathology. Using a detailed FE model of the rat brain, Green-Lagrange strain and strain rate correlated spatially and in magnitude with MRI and histopathology measures of diffuse axonal and glial injuries 14 days after the impact (Donat et al., 2020). Microglial response and axonal injury often co-localise in animals and humans and can still be detected several years after an injury (Csuka et al., 2000; Lafrenaye et al., 2015; Scott et al., 2015), possibly contributing to tau accumulation in cases of CTE (Cherry et al., 2016).

In humans, we have used a Finite Element (FE) model of traumatic brain injury (TBI) which incorporated detailed anatomy of the brain to predict the distribution of Green-Lagrange strain and strain rate within the brain during an American Football head collision, a fall and a motorcycle accident (Ghajari et al., 2017). In all cases, the model predicted high levels of strain within the depths of sulci, where the CTE pathology has been seen (McKee et al., 2016), and converged with where abnormalities in MRI metrics of axonal injury were observed in a large cohort of TBI patients. These findings replicated earlier observations whereby the introduction of sulci to computational models resulted in strain reducing in non-sulcal areas and areas of high strain localising below the sulci (Ho and Kleiven, 2009). However, using a single American Football case limits the implications of the findings to large cohorts of American Football collisions, particularly that players are exposed to different head impacts based on their playing position.

Playing at different positions has been shown to influence risk of concussion in American footballers (Dick et al., 2007; Nathanson et al., 2016; Pellman et al., 2004). This is likely due to the fact that head kinematics, such as linear and rotational accelerations of the head, and brain deformation vary depending on the position of the player (Funk et al., 2012; Karton et al., 2020). Brain deformation has been shown to be particularly sensitive to rotational acceleration (Gennarelli et al., 1972; Gennarelli et al., 1987; Holbourn, 1943; Kleiven, 2007; Margulies et al., 1990; Post and Blaine Hoshizaki, 2015; Takhounts et al., 2013). Therefore, playing a position where there is high risk of head impacts with high rotational acceleration may increase a player’s risk of concussion. However, it is unclear whether playing at different positions will also change the pattern of force distribution across the brain and particularly in sulcal depths, a location of CTE pathology.

The present study addresses this question. We used our previously developed FE model of TBI to simulate 148 American Football head impacts and predict strain and strain rate across the brain during impacts. We divided our cohort based on their playing position into three profiles and tested whether playing position is a factor in determining the pattern and severity of mechanical strain and strain rate across the whole brain and particularly in sulci. Playing at positions where the head is exposed to larger rotational impacts is likely to increase strain and strain rate in sulci, so we also tested whether rotational head kinematics can predict the sulcal strain and strain rate across all collisions.

## 2 Methods

### 2.1 Video analysis and laboratory reconstructions

**Fig. 1:**
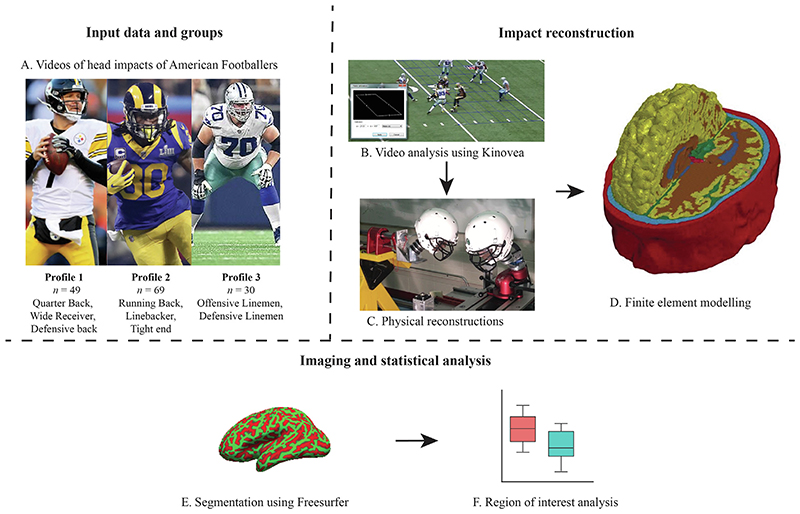
Study overview. (A) Player positions in each profile, (B) Snapshot of the video analysis software, Kinovea, used to determine the speed and angle of colliding players, (C) Lab setup for physical reconstruction of the collisions, (D) The finite element model of TBI loaded by the head accelerations recorded in the lab tests, (E) Freesurfer segmentation of the sulcal and gyral regions applied to the computational predictions of strain and strain rate, (F) Statistical analysis of strain and strain rate distribution in sulci and gyri.

Videos of 32 American football games were analysed using previously described methodology (Fig. 1) (Karton et al., 2020). In brief, players were grouped according to their positions and separated into 3 different profiles based on impact frequency, strain magnitude and time interval (Fig. 1A) (Karton et al., 2020). Recorded footage was used to document all head contact and estimate impact velocity, location/orientation, and event type using Kinovea software (version 0.8.20) (Fig. 1B – see supplementary materials for more information on the validation of the video analysis method).

166 exemplar events were selected for reconstruction to represent the trauma profile of the 3 distinct groups. Laboratory reconstructions were direct reconstructions from videos of the exemplar events. Exemplar events were selected for reconstruction for each permutation of player position, head-impact location (helmet, shoulder, hip and ground to helmet) and velocity level (collisions: very low = <2.0 m/s, low = 2.1–4.5 m/s, moderate = 4.6–7.0 m/s, high = 7.1–9.5 m/s, very high = 9.6 + m/s; Falls: very low = <2.0 m/s, low = 2.1–4.0 m/s, moderate = 4.1–6.0 m/s, high = 6.1–8.0 m /s, very high = 8.1 + m/s) to build a pool of impact events representing all impact conditions. The current analysis excluded events that represented redundant conditions in the previous analysis by (Karton et al., 2020). Exemplar events were not selected on the basis of concussion occurring, but represented a range of impacts experienced by players in a typical play.

Parameters obtained from video analysis were used to guide physical reconstructions of the head impact events using a Hybrid III 50th-percentile adult male headform and neutral unbiased neckform (Walsh et al., 2018), with mounted accelerometers capturing linear and rotational acceleration of the headform (Fig. 1C). 18 events were discarded at the simulation stage due to incorrect coding of velocity leaving 148 impacts.

### 2.2 Finite element model and injury simulations

A 3D finite element model of the human head developed at Imperial College London was used to calculate brain deformations from each head impact. The model was developed using a high-resolution T1-weighted magnetic resonance image of a healthy 34-year-old male participant and consists of nearly one million hexahedral elements and a quarter of a million quadrilateral elements, representing 11 tissues, including the scalp, skull, brain, meninges, subarachnoid space and ventricles, as well as anatomical features such as sulci. Model material characteristics, development and validation details are described in previous work (Ghajari et al., 2017) and supplementary materials.

The head model was loaded by linear and rotational accelerations obtained from the physical reconstructions of the impacts. For each element of the model, we determined what we call the strain hereafter, which is the maximum principal value of the Green-Lagrange strain tensor and a measure of how much the element has been stretched with respect to its original shape. In addition, we determined the strain rate, which is the highest value of the total time derivative of the Green-Lagrange strain tensor that the element experienced during the simulation and is a measure of the maximum time rate of stretch within each element (Holzapfel, 2000). Values for strain and strain rate were encoded in an element-by-element basis and saved as a NIFTI (Neuroimaging Informatics Technology Initiative) file for neuroimaging analysis.

### 2.3 Injury model image processing and analysis

Strain and strain rate data for each impact were analysed using fMRIB Software Library (FSL) v6.0.2. The 90th-percentile maximum principal strain and strain rate across the whole brain was calculated per impact to account for possible abnormally large strains in poorly defined elements and used to represent the peak strain and strain-rate values reached across the whole brain during and impact. The T1-weighted magnetic resonance image used to generate the computational model was segmented using Freesurfer (Fischl et al., 2004). This process resulted in an accurate reconstruction of the pial surface and white matter–grey matter boundary as a 3D surface for each of the left and right hemispheres within a conformed Freesurfer space (Desikan et al., 2006). The Freesurfer surface was then subdivided into regions of interest based on the Destrieux Atlas (Fischl et al., 2004) and anatomical labelling resulting in regions of interest for 30 gyri and 33 sulci in each hemisphere (Ghajari et al., 2017). The NIFTI images containing strain and strain rate values were registered to Freesurfer space using a standard affine transformation. The mean value of the maximum principal strain and strain rates were then calculated for areas within each region of interest mask generated from the Freesurfer segmentation. These values were further averaged to calculate a mean strain and strain rate for gyral and sulcal regions for each impact. To investigate the relationship between impact kinematics and predicted brain deformation as modelled, we compared the peak resultant linear and rotational velocity and accelerations of the head in each impact with the 90th percentile strain and strain rate across the whole brain.

### 2.4 Statistical tests

Statistical tests were completed in the open source software package R. Two-tailed paired T-test was performed to compare whether mean strain and strain rate was higher in gyri or sulci and Cohen’s d reported as a measure of the effect size. In cases where the normality assumption was violated, the Wilcoxon test was used. A one-way ANOVA reported as F-value (degrees of freedom of independent variables, residuals), p-value, was used to assess the effect of player position profile (one level, three factors) on peak linear and rotational acceleration and velocity, 90th percentile strain across the whole brain and 90th percentile strain rate across the whole brain. A two-way repeated measures ANOVA using player position profile (one level, three factors) and brain structure as a within subject factor (one level, two factors: gyri and sulci) was conducted comparing the profiles to explore profile-wise differences in strain and strain rate in sulci and gyri. Values for strain were root or log-transformed to fit assumptions for normality. Pearson correlation was used to determine the relationship between sulcal strain and strain rate with peak resultant linear and rotational velocity/acceler-ation. Multiple linear regression analysis was used using peak linear and rotational acceleration and velocity as predictor variables to test the contributions of the predictor variables in predicting 90th percentile strain and strain rate in the sulci.

## 3 Results

### 3.1 There are distinct differences in strain and strain rate across the whole brain and head kinematics between players at different positions

Comparing whole brain 90th percentile strain and strain rates across the different player profiles, a one-way ANOVA indicated there was a significant effect of player position profile on both strain (F_2,145_ = 4.401, *p* = 0.014) and strain rate (F_2,145_ = 4.672, *p* = 0.011). Post-hoc Tukey HSD tests showed that these effects were driven by significant differences between profile 1 and profile 3 player impacts for strain (*p* = 0.016) and strain rate (*p* = 0.012) (Table 1).

We then investigated if there were differences in head impact kinematics between players of different positions. A one-way ANOVA showed a significant effect of player position profile on peak resultant linear acceleration (F_2,145_ = 3.887, *p* = 0.023), linear velocity (F_2,145_ = 4.241, *p* = 0.016), rotational acceleration (F_2,145_ = 4.583, *p* = 0.012) and rotational velocity (F_2,145_ = 4.574, *p* = 0.012) (Table 1). Post-hoc Tukey HSD tests showed that these effects were driven by higher values in linear acceleration (*p* = 0.026), linear velocity (*p* = 0.023), rotational acceleration (*p* = 0.028) and rotational velocity (*p* = 0.022) in profile 1 impacts compared to profile 3. Profile 1 impacts additionally had higher rotational acceleration (*p* = 0.028) and rotational velocity (*p* = 0.035) when compared to profile 2 impacts. All other comparisons were not significant.

### 3.2 Larger strain is present in sulcal regions of all players

**Fig. 2:**
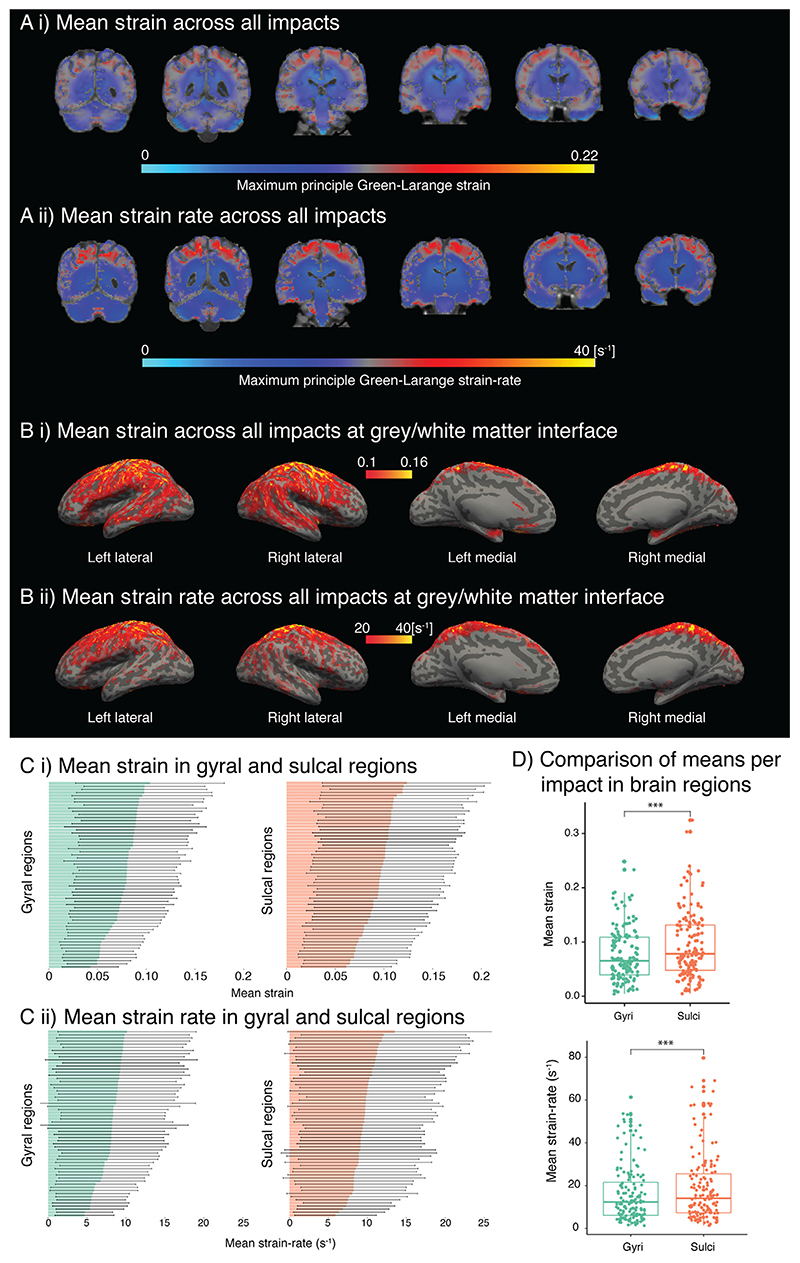
Computational results for all players. (A) Whole brain mean value of strain and strain rate (areas in yellow–red indicate higher values and light blue-blue lower values), (B) Mean value of strain and strain rate at grey/white matter interface (sulci: light grey, gyri: dark grey, yellow areas indicate higher values), (C) Mean value and standard deviation of strain and strain rate in each sulcal and gyral region, and (D) Mean values collapsed to two anatomical regions, gyri and sulci. (For interpretation of the references to colour in this figure legend, the reader is referred to the web version of this article.)

Large strain and strain rate were confined to the depths of sulci in a symmetrical distribution, with lower strain and strain rate seen at the cortical surface and gyral regions and noticeably in sub-cortical structures (Fig. 2A). We confirmed the distinction between the maximal strain and strain rate in the sulci and gyri by mapping out these fields at the grey–white matter interface (Fischl et al., 2004; Fischl, 2012) and looking at average strain maps (Fig. 2B). We further analysed this data by determining strain and strain rate in sulcal and gyral regions across all impacts and comparing them (Fig. 2C). We found significantly higher strain (0.09 ± 0.06 vs 0.08 ± 0.05, V = 3, *p* < 0.001, *d* = 1.31) and strain rate (19 ± 16/s vs 16 ± 13/s, V = 84, *p* < 0.001, *d* = 0.89) in the sulcal regions compared to gyral regions (Fig. 2D).

### 3.3 There is a consistent spatial pattern of higher strain and strain rate in sulci than gyri in players at different positions, and largest sulcal strain and strain rate in players at position profile 1

**Fig. 3:**
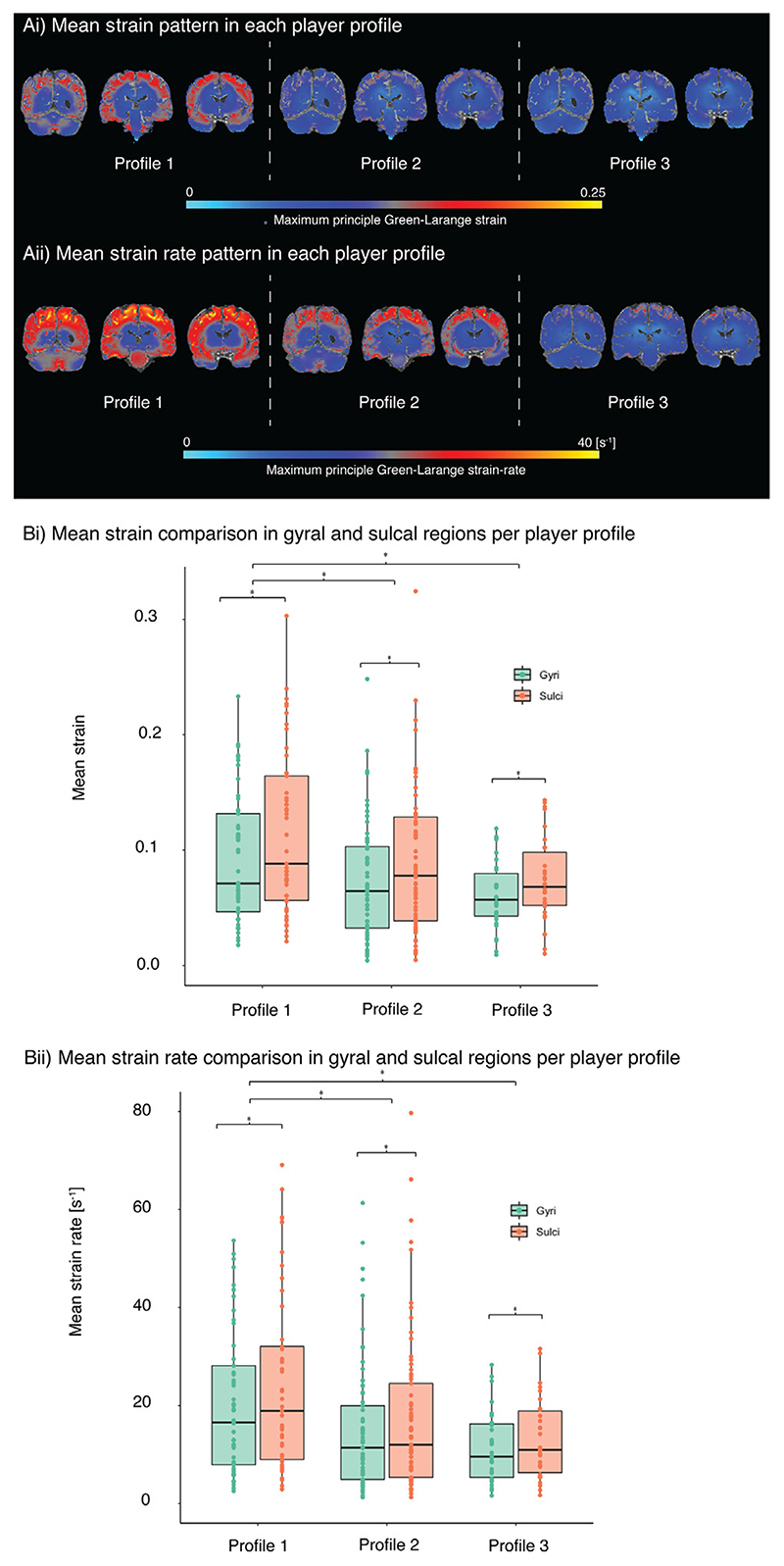
Predicted patterns of cortical strain in players at different positions. (Ai) Mean value of strain and (Aii) strain rate across the whole brain, (Bi) comparison between mean strain and (Bii) strain rate across profiles and sulci/gyri.

We further investigated if the sulcal and gyral differences present across all impacts were preserved between player profiles and whether player position had an effect on increasing sulcal strain and strain rate. Brain contours of strain showed that strains and strain rates are larger in the brain of players at position profile 1 compared to the other two position profiles and concentrated in sulcal regions (Fig. 3A). A repeated measures ANOVA showed that there was a significant effect of player position profile (F_2,145_ = 4.26, *p* = 0.016) and brain region (sulcal vs gyral, F1,145 = 718, *p* < 0.001) on strain in sulci and gyri regions. Furthermore, there was an interaction between player position profile and brain region on strain (F = 3.63, *p* = 0.029). Post-hoc Tukey HSD tests showed that these effects were driven by higher strain in player position profile 1 compared to profiles 2 and 3 in both gyri (profile 1 vs 2: *p* = 0.047, profile 1 vs 3: *p* = 0.027) and sulci (profile 1 vs 2: *p* = 0.003, profile 1 vs 3: *p* < 0.001) with a more significant effect of player position profile on strain in sulci than gyri (Fig. 3Bi).

A similar effect was found when investigating strain rate, with a significant effect of player position profile (F_2,145_ = 4.45, *p* = 0.013) and brain region (sulcal vs gyral, F1,145 = 1109.82, p < 0.001). There was no interaction between player position profile and brain region on strain rate. Post-hoc tests showed that these effects were driven by higher strain rates in player position profile 1 compared to profiles 2 and 3 in both gyri (profile 1 vs 2: *p* = 0.039, profile 1 vs 3: *p* = 0.026) and sulci (profile 1 vs 2: *p* = 0.0016, profile 1 vs 3: *p* < 0.001) (Fig. 3Bii).

### 3.4 There is a strong correlation between head rotational velocity and acceleration and strain and strain rate in sulci

Finally, we investigated whether there is correlation between measures of head kinematics, namely peak values of linear velocity, linear acceleration, rotational velocity and rotational acceleration, and strain and strain rate in sulci. We found significant positive correlations between all head kinematics measures and strain and strain rate in sulci (*p* < 0.001) (Table 2). R^2^ values were higher for rotational acceleration and velocity than linear acceleration and velocity. Regression analysis incorporating linear and rotational velocity and acceleration indicated rotational velocity (*p* < 0.001, β = 3.03, 95% CI 2.39 – 3.65) and acceleration (*p* < 0.001, β = 22.26, 95% CI 17.02 – 27.50) were both significant independent predictors of strain in sulci while linear velocity and acceleration were not. In predicting strain rate in sulci, rotational velocity (*p* = 0.01, β = 206.77, 95% CI 46.65 – 366.89) and rotational acceleration (*p* < 0.001, β = 8160.87, 95% CI 6828.41 – 9493.32) were significant independent predictors while all other factors were non-significant.

## 4 Discussion

We showed that the player position has a significant effect on increasing strain and strain rate in the sulci, a prominent location of the pathology in the neurodegenerative disease CTE (McKee et al., 2016) and where white matter abnormalities in a larger cohort of TBI patients have been observed (Ghajari et al., 2017). This was allowed by using a finite element model of TBI which incorporates fine details of brain anatomy including sulci. This model allowed us to predict strain and strain rate in the sulci from the head kinematics measurements made in lab reconstructions of the American Football collisions. By using this approach, we were also able to show a strong correlation between rotational motion of the head and sulcal strain and strain rate.

We found that strain and strain rate were highest in sulci for players in position profile 1 (defensive back, quarter back and wide receiver). Players at this position were exposed to the largest linear and rotational accelerations and velocities. Previous work has shown that players at this position are usually exposed to high magnitude forces, but at low frequency, and they report highest incidence of concussions (Dick et al., 2007; Horstemeyer et al., 2019; Karton et al., 2020; Nathanson et al., 2016). These results are in keeping with previous animal studies which demonstrated that high strain in the cortex is required to produce concussion-like symptoms (Tagge et al., 2018). Conversely, players in position profile 3 (defensive linemen and offensive linemen), who experience head impacts with low magnitude but at high frequency, had the lowest magnitude of strain within the sulci. In contrast to concussion, there is limited work that has investigated the link between player position and risk of developing CTE. A recent histopathological study of deceased footballers could not find an association between player position and risk of developing CTE (Mez et al., 2020).

There is little consensus as to the frequency or severity of injuries required to produce CTE pathology. Previous studies have shown that CTE like pathology can be produced from single severe head injuries (Johnson et al., 2012; Smith et al., 2013). However, the majority of cases, particularly in a sporting context, have been in relation to repeated mild TBIs (McKee et al., 2009) or what is described as exposure to subthreshold impacts (Bieniek et al., 2015; McKee et al., 2014; Stein et al., 2015) which are distinct from concussions as no immediate symptoms are produced. In animal modelling, CTE pathology can be produced when the brain is exposed to lower strains than strains required to produce concussion like symptoms (Tagge et al., 2018). It is likely that there is an interaction between brain deformation and impact frequency in the risks associated with developing CTE, with different mechanisms in player groups who are exposed to either low frequency, higher amplitude impacts or high frequency, lower amplitude impacts (Horstemeyer et al., 2019).

The mechanistic link between impact frequency, brain strain and pathology is unclear. Previous work has implied that brain strain can directly contribute to long term clinically relevant pathology. Mechanical strain can cause blood–brain barrier injury (Shreiber et al., 1997; Shreiber et al., 1999), which may lead to the typical pattern of tau pathology observed in the sulci along small blood vessels. Sulcal strain also corresponds with where disruption to white matter axons have been observed in cases of CTE (Holleran et al., 2017; McKee et al., 2013). Clinically, disruption to the white matter has been proposed as a key contributor to the production of short and long term deficits after brain injury (Sharp and Jenkins, 2015). In a recent study that combined fidelity finite element modelling with MRI and quantitative histopathology in a rat model of TBI, a clear relationship was found between strain and post-traumatic brain pathology, including white matter disruption and neuroinflammation (Donat et al., 2020). This damage can persist several years after an injury (Scott et al., 2015) and has been shown to possibly contribute to tau accumulation in cases of CTE (Cherry et al., 2016).

We replicate findings from our previous study, whereby in 3 cases, including one American Football impact, higher strains were found in the sulci compared to gyri (Ghajari et al., 2017). Similar observations were made by an earlier study (Ho and Kleiven, 2009) when introducing sulci into a finite element model, suggesting these results cannot be artefacts of a single model. We extended these studies by simulating a large number of American Football head collisions, which confirmed the same location of high strain concentrations. Moreover, consistently across the three position profiles, the observed spatial patterns of maximal deformation were replicated, with strain and strain rate highest in sulci than gyri. While the effect sizes of these differences between sulci and gyri are large, there is no evidence to suggest that the differences alone are sufficient to produce pathology preferentially in one area than another. It is likely that impact frequency needs to be considered. Future work is required in in vivo or in vitro models of TBI to elucidate the relationship between impact frequency, brain deformation and production of tau pathology.

Kinematic magnitudes are closely related to brain deformations (Post and Blaine Hoshizaki, 2015). We found that linear and rotational measures of head kinematics during an impact were highly correlated with the strain and strain rate in sulci. However, when all of these kinematics measures were incorporated into a model, only rotational acceleration and velocity predicted strain and strain rate. This is in keeping with suggestions that rotational acceleration plays a key role in producing high tissue deformation and shearing within the brain (Meaney and Smith, 2011; Post et al., 2017) and has been demonstrated consistently across a variety of sports (Forero Rueda et al., 2011; Post and Blaine Hoshizaki, 2015; Rowson and Duma, 2013). With our modelling approach, we were able to show the link between rotational kinematics of the head and larger forces in sulci. Therefore, putting in place measures to mitigate exposure to large rotational forces is likely to reduce the effects of head collisions on producing brain injuries.

The limitations of the video analysis method and impact reconstructions used in the present study have been previously described (Ghajari et al., 2017; Karton et al., 2020) and within the supplementary materials, but these methods still remain one of the key approaches to determining the kinematics of the head during sports collisions. We used an unbiased neckform which has been shown to be repeatable and typically gives very small standard deviations on series of impacts to various locations (Chen et al., 2020; de Grau et al., 2020; Post et al., 2020). The unbiased neck has been shown to perform similarly to the Hybrid-III neck (Walsh et al., 2018). The Hybrid-III performs within the wide corridor of responses in terms of stiffness observed for the human neck, and we have observed minimal influences of neck stiffness on brain strains in helmeted impacts (Rousseau et al., 2010). However, there are known limitations, such as its stiffness in axial loading (Yoganandan et al., 1989). Presently, these limitations have yet to be addressed in available neckforms, and is in part why American Football helmets are currently tested universally using the Hybrid-III neck (Funk et al., 2020). This limitation may be addressed in the future when an improved neck is available. We were limited to the selection of only a single exemplar event per condition. Future work should be extended to using multiple events per condition to generate a more reliable database of impacts representing the exposure of players in normal play. We are also limited by the lack of a measure of impact frequency, which limits our interpretation of the risk of developing CTE for players of different positions based on single impact deformations. Future studies should therefore incorporate a measure of a cumulative load. Additionally, there is a risk of bias against profile 3 conditions due to the lower number of impacts included within our dataset.

In summary, we found that player position in American Football affects the magnitude of strain and strain rate across the brain and particularly in sulci. This is likely due to the effect of player position on impact kinematics. The large strain and strain rate may explain the higher rate of concussion in offensive and defensive linemen positions. However, it is likely that the impact frequency is a key factor in producing the neurodegenerative processes at lower strains than those required to produce concussion like symptoms. This is a limitation of the study, which needs to be addressed in future when more information is available on the repetitive effects of forces on primary and secondary progressive damage to the brain. We also found that rotational head motion is a key determinant in producing large strains in the sulci. This work can contribute to the development of protective, preventative and injury management strategies aimed at reducing both acute and long-term effects of head collisions in sports.

## Supplementary Material

Supplementary materials

## Figures and Tables

**Table 1:** Mean kinematics and brain deformation per player position profile.

Kinematics and brain deformation measures	Profile 1 *n* = 49	Profile 2 *n* = 69	Profile 3 *n* = 30
90th Percentile Strain	0.16 ± 0.10	0.13 ± 0.09	0.11 ± 0.05
90th Percentile Strain rate (s^–1^)	35 ± 28	26 ± 25	20 ± 13
Linear Velocity (m/s)	3.9 ± 2.2	3.1 ± 1.7	2.8 ± 1.5
Linear Acceleration (m/s^2^)	428 ± 295	329 ± 250	273 ± 196
Rotational Velocity (rad/s)	25 ± 0.012	19 ± 0.012	18 ± 0.009
Rotational Acceleration (krad/s^2^)	3 ± 0.0019	2 ± 1.6	2± 1.2

**Table 2:** Pearson correlation between mean sulcal strain and kinematics measurements of head collisions.

	Linear Velocity	Linear Acceleration	Rotational Velocity	Rotational Acceleration
Strain(r^2^, 95% CI)	0.69 (0.59–0.77)	0.70 (0.60–0.77)	0.89 (0.86–0.92)	0.88 (0.84–0.91)
Strain Rate (r^2^, 95% CI)	0.75 (0.67–0.81)	0.78 (0.71–0.84)	0.83 (0.77–0.87)	0.93 (0.91–0.95)

## Data Availability

Raw and processed physical data, and game video clips are currently being stored at the University of Ottawa, Canada. Computational data is stored at Imperial College London. Data is available upon reasonable request.
