# Supplementary material for: Player position in American football influences the magnitude of mechanical strains produced in the location of chronic traumatic encephalopathy pathology: A computational modelling study
Source: J Biomech. Author manuscript; Available in PMC 2022 Feb 9. (PMC7612336; doi:10.1016/j.jbiomech.2021.110256)
Supplement: Supplementary materials [file EMS141009-supplement-Supplementary_materials.docx]

**Supplementary materials**

**S1. Video analysis**

The video analysis method was validated using real-world ice hockey collisions. The average error reported in the estimated collision speed and angle was approximately 10% (Post et al., 2018). The factors that influence this error were documented as the area of the calibration field, the proximity of the calibration field to the impact event, and the orientation of the impact relative to the camera view. Based on the reported sources of error, it is likely that the error in the reconstructions of this study would be equivalent or less. The field in American football contains more field markers that an ice hockey rink. This increases the likelihood that the calibration field can be set directly at the site of impact, or within close proximity. These reconstructions were of professional level football where the camera placements are many and typically perpendicular to the field, thus increasing the field of view and accuracy of the measurement.

**S2. The finite element model of TBI**

We used an image-based mesh generation approach to develop the head FE mesh, similar to previous work (Chen and Ostoja-Starzewski, 2010; Ho and Kleiven, 2009). Briefly, we segmented the T1-weighted image of the subject into skin, skull, CSF, grey matter, white matter, ventricles and brain stem using Freesurfer and FSL (Dale et al., 1999; Fischl et al., 2004). Each voxel of the image was replaced with a hexahedral element (1.5mm edge length) using an in-house code. The falx and tentorium were added between the hemispheres and cerebrum/cerebellum respectively using shell elements. A layer of shell elements was added on the grey matter to represent the pia matter. Figure 1D shows the FE mesh of the model and a close-up view of a sulci. The mesh at the interface between CSF/skull and grey matter/CSF was smoothed using the algorithm explained in (Chen and Ostoja-Starzewski, 2010). However, we did not smooth the mesh at the interface between grey matter and white matter because they had the same material properties. The final model had an average element size of 1.5mm, with 0.1mm for the smallest element. The initial stable time step was 720 nanoseconds, which was dictated by the CSF. 90% of the elements of the brain had an aspect ratio smaller than 3 and 87% had a Jacobian larger than 0.5, which indicate a good quality for the vast majority of the mesh. The mesh quality at the grey/white matter interface, which is the region of interest in this study, had a particularly good quality because the mesh was not smoothed at this interface.

**S3. Evaluation of the TBI FE model’s prediction of brain displacement against rotational cadaver experiments**

The model predictions of brain displacement were compared to recently published experimental data from well-documented and open-access post-mortem human subject (PMHS) experiments (Alshareef et al., 2018). Controlled rotational motion was applied to the PMHS head and the displacement of 24 receiving crystals inserted in the brain were measured using the sonomicrometry method (Alshareef et al., 2020). From these experiments, we simulated four tests, resulting in 288 displacement time histories for comparisons. We used the CORA analysis, which show that the majority of the displacement predictions have fair to excellent fidelity (CORA>0.44).

We simulated tests on subject IDs 846 and 900. The length, breadth and height of the model were scaled to match the dimensions of the subjects. The centre of gravity (CoG) was determined using the method explained in (Alshareef et al., 2020). We simulated coronal and sagittal motions with 40rad/s peak rotational velocity and 30ms duration. The rigid skull of the model was loaded by the three components of the linear acceleration and three components of the rotational velocity measured during the experiments at the centre of gravity. The displacements of the receiver nodes were recorded with respect to the head axes fixed at the CoG.

Figures S1-4 show the components of PMHS receiver displacements overlaid on the predicted displacements. Good agreement can be observed between predictions and experiments for a number of receivers and axes. The CORA analysis based on (Gehre et al., 2009) and the settings suggested in (Giordano and Kleiven, 2016) were used to compare the pulses. Figure S11 confirms that a majority of the displacement predictions have fair to excellent fidelity. A few predictions have marginal or unacceptable fidelity, which warrant further investigation. The mean CORA for all tests and axes range from 0.50 to 0.65, which indicates an overall fair fidelity of the predictions.


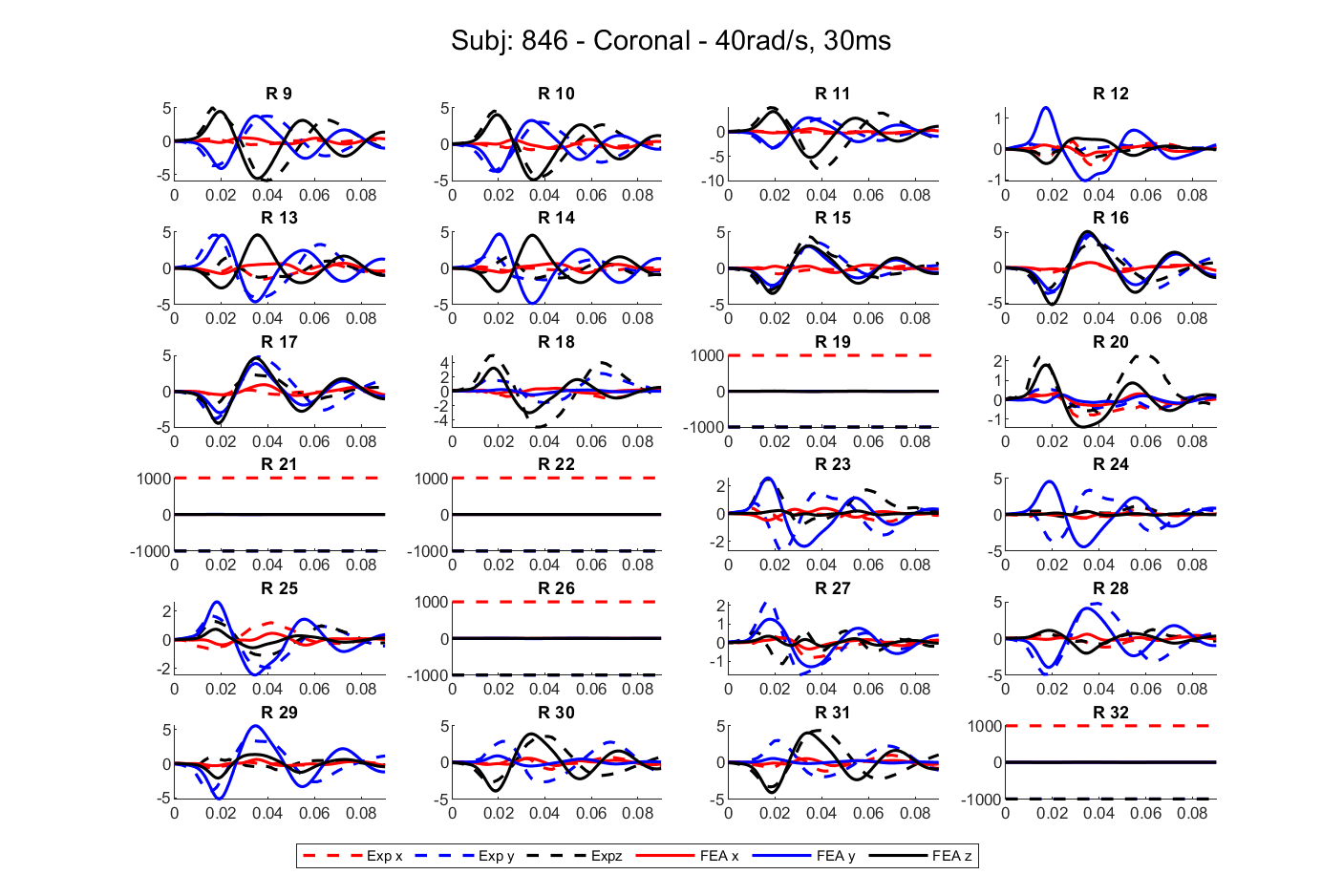


**Figure S1.** Relative brain/skull displacements at crystal receivers from the PMHS experiment (subject 846, coronal rotation) and predicted by the TBI model. The PMHS displacement data were not available for receivers R 19, 21, 22, 26 and 32. Reproduced from (Fahlstedt et al., 2021)


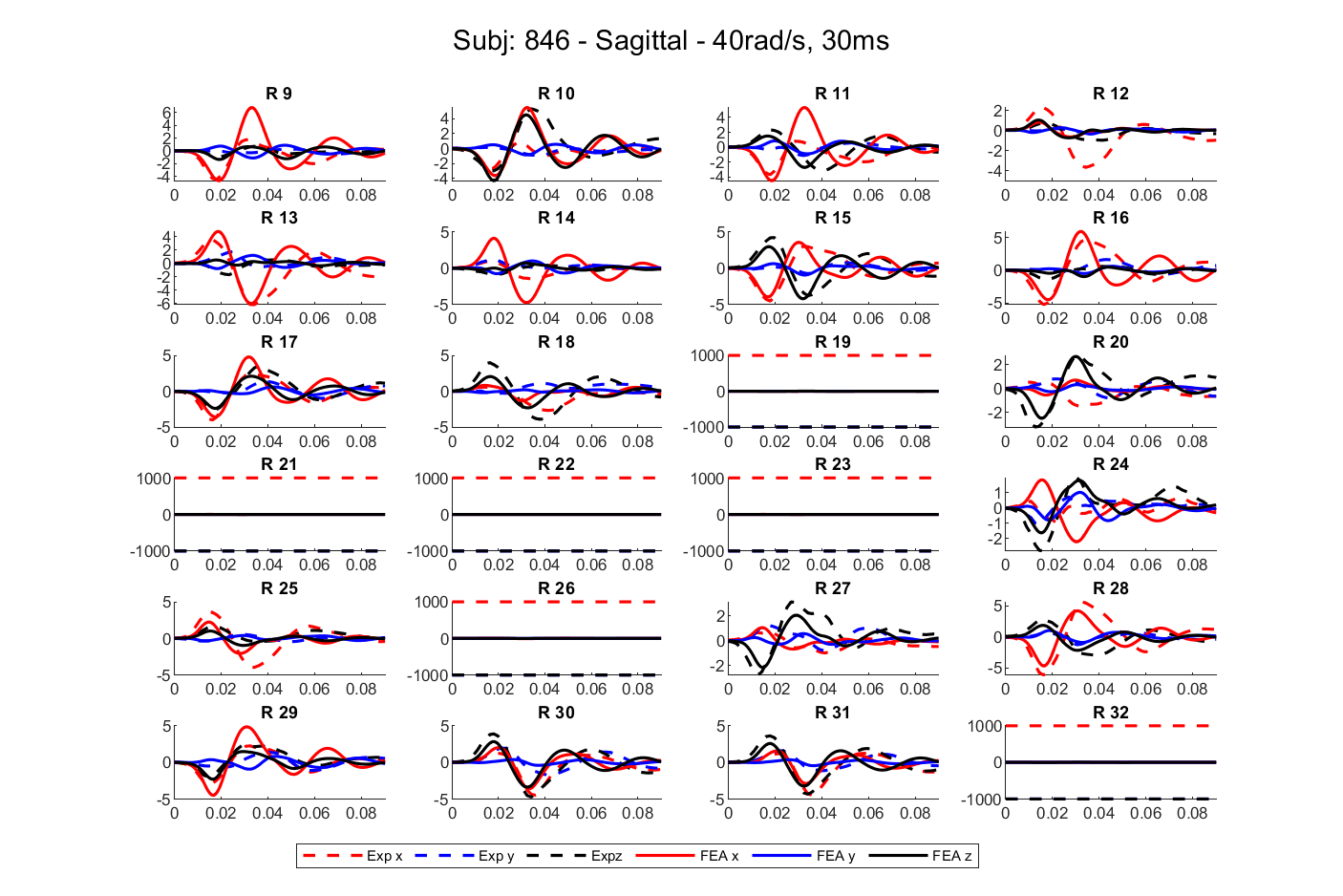


**Figure S2.** Relative brain/skull displacements at crystal receivers from the PMHS experiment (subject 846, sagittal rotation) and predicted by the TBI model. The PMHS displacement data were not available for receivers R 19, 21, 22, 26 and 32. Reproduced from (Fahlstedt et al., 2021)


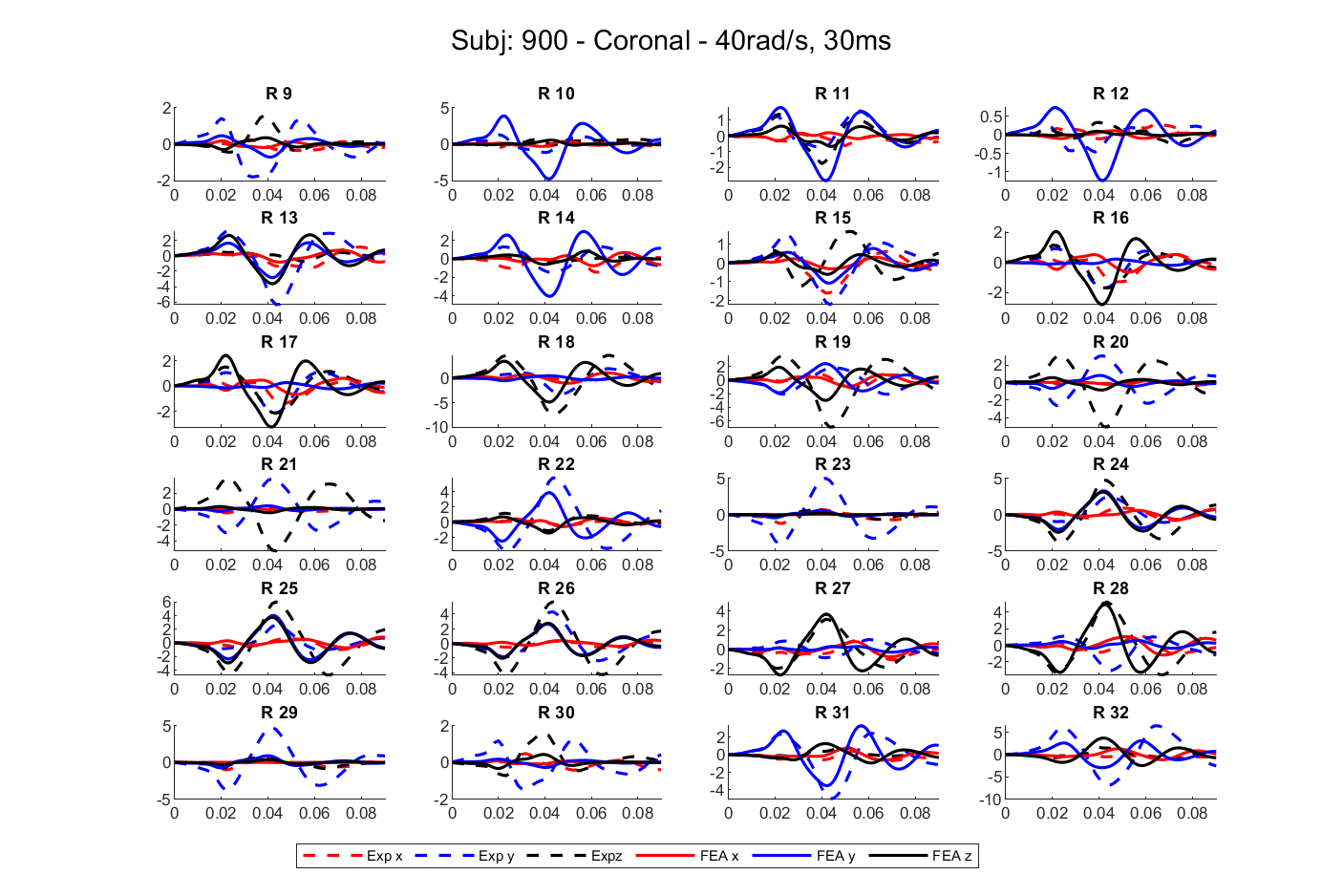


**Figure S3.** Relative brain/skull displacements at crystal receivers from the PMHS experiment (subject 900, coronal rotation) and predicted by the TBI model. Reproduced from (Fahlstedt et al., 2021)


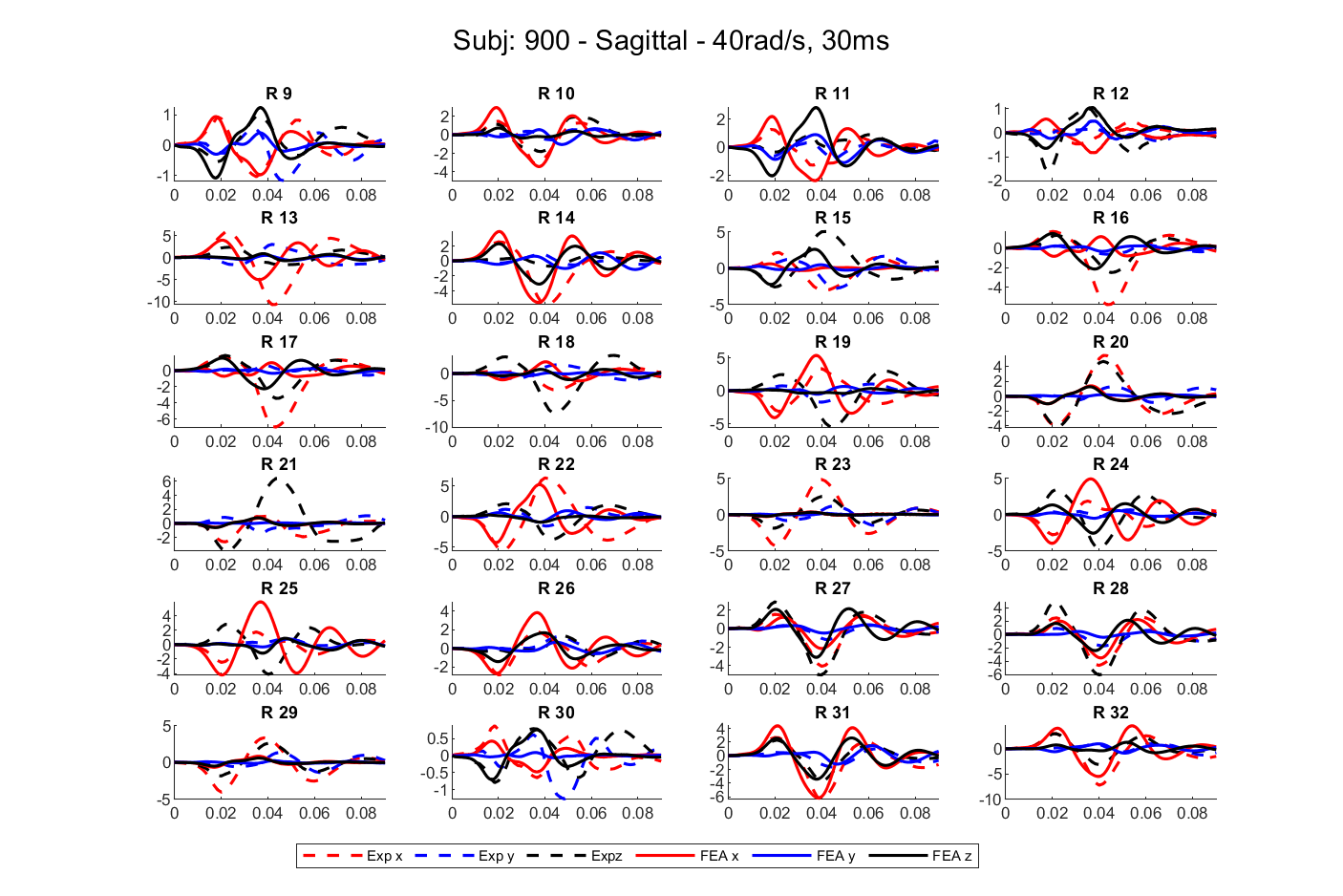


**Figure S4.** Relative brain/skull displacements at crystal receivers from the PMHS experiment (subject 900, sagittal rotation) and predicted by the TBI model. Reproduced from (Fahlstedt et al., 2021)

| 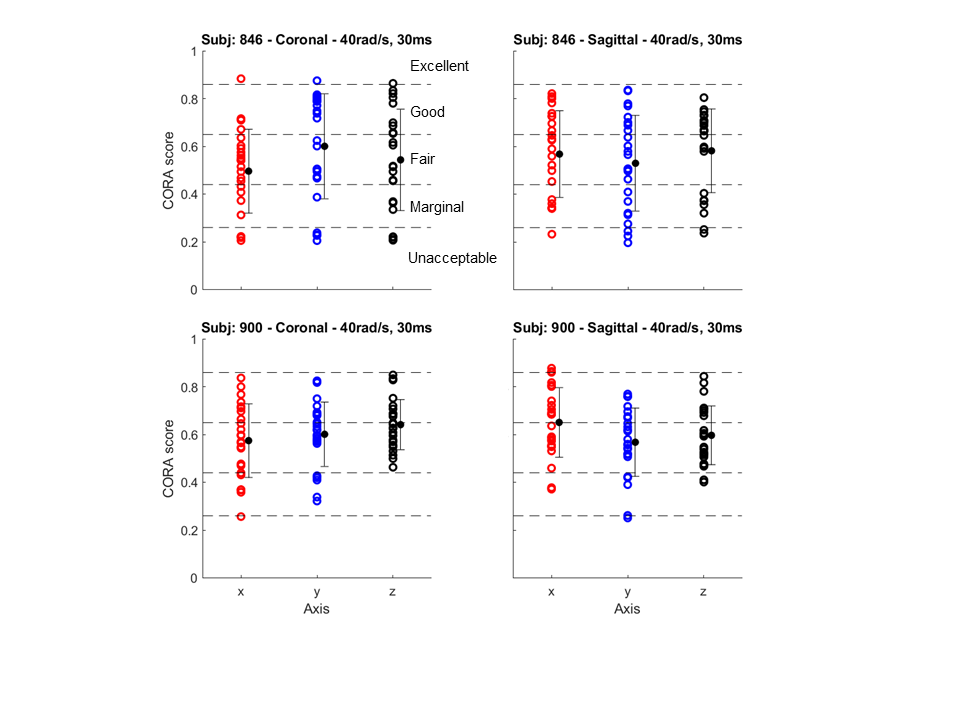 |
| --- |

**Figure S5.** CORA scores. The broken lines indicate the borders of biofidelity classifications (unacceptable, marginal, fair, good and excellent) defined in (ISO-TR 9790, 1999). Reproduced from (Fahlstedt et al., 2021)

**S6. Limitations of Finite Element modelling**

The accuracy of our finite element model of TBI, as with any other models, depends on a number of factors including the mesh density and quality, fidelity of the anatomy and fidelity of material models and properties. The small element size used in this model allowed us to preserve key anatomical features, particularly sulci, and obtain a reasonable stable time step for explicit simulations. This element size is slightly larger than that used in previous FE models that incorporated sulci and gyri (1.5 vs 1mm) (Chen and Ostoja-Starzewski, 2010; Ho and Kleiven, 2009). Future work may study the effects of refining the mesh on the predictions of strain and strain rate in sulci. Since an image-based meshing approach was used, the majority of the elements, particularly those within the brain and at grey/white matter interface, had good quality. We incorporated the nonlinear and rate sensitive mechanical response of the brain using a visco-hyperelastic material model with properties obtained from experiments on post-mortem human tissue (Kleiven, 2007). Mechanical properties of the tissue are likely to change post-mortem but currently there is no other method that can characterise the mechanical response of the human brain in vivo at large strain and high strain rates. In order to build confidence in the predictions of the model, we evaluated the performance of the model in predicting the displacement of discrete points across the brain by making comparison with PMHS data. Although our results show fair to excellent fidelity of the model in predicting displacement, a more ideal evaluation contains comparison with experimentally measured strain and strain rate. There is limited previous work that have reported strain in PMHS tests (Hardy et al., 2007), and they calculate the strain from the displacement of a discrete cluster of points in the brain. With the availability of more high quality PMHS experimental data that contain more accurate measurements of strain and strain rate, the model validation should be extended in future.

**References**

Alshareef, A., Giudice, J.S., Forman, J., Salzar, R.S., Panzer, M.B., 2018. A Novel Method for Quantifying Human In Situ Whole Brain Deformation under Rotational Loading Using Sonomicrometry. Journal of neurotrauma 35, 780-789.

Alshareef, A., Giudice, J.S., Forman, J., Shedd, D.F., Reynier, K.A., Wu, T., Sochor, S., Sochor, M.R., Salzar, R.S., Panzer, M.B., 2020. Biomechanics of the Human Brain during Dynamic Rotation of the Head. Journal of neurotrauma 37, 1546-1555.

Chen, Y., Ostoja-Starzewski, M., 2010. MRI-based finite element modeling of head trauma: spherically focusing shear waves. Acta Mechanica 213, 155-167.

Dale, A.M., Fischl, B., Sereno, M.I., 1999. Cortical surface-based analysis. I. Segmentation and surface reconstruction. Neuroimage 9, 179-194.

Fahlstedt, M., Abayazid, F., Panzer, M.B. *et al.* Ranking and Rating Bicycle Helmet Safety Performance in Oblique Impacts Using Eight Different Brain Injury Models. *Ann Biomed Eng* (2021). https://doi.org/10.1007/s10439-020-02703-w

Fischl, B., van der Kouwe, A., Destrieux, C., Halgren, E., Segonne, F., Salat, D.H., Busa, E., Seidman, L.J., Goldstein, J., Kennedy, D., Caviness, V., Makris, N., Rosen, B., Dale, A.M., 2004. Automatically parcellating the human cerebral cortex. Cereb Cortex 14, 11-22.

Gehre C., Gades H., Wernicke P. (2009). Objective Rating of Signals Using Test and Simulation Responses. Proceedings of the 21st International Technical Conference on the enhanced Safety of Vehicles ESV (Paper 09-0407), Stuttgart (Germany).

Giordano, C., Kleiven, S., 2016. Development of an Unbiased Validation Protocol to Assess the Biofidelity of Finite Element Head Models used in Prediction of Traumatic Brain Injury. Stapp Car Crash J 60, 363-471.

Hardy, W.N., Mason, M.J., Foster, C.D., Shah, C.S., Kopacz, J.M., Yang, K.H., King, A.I., Bishop, J., Bey, M., Anderst, W., Tashman, S., 2007. A study of the response of the human cadaver head to impact. Stapp Car Crash J 51, 17-80.

Ho, J., Kleiven, S., 2009. Can sulci protect the brain from traumatic injury? J Biomech 42, 2074-2080.

ISO/TR 9790. The International Organization for Standardization (ISO). Road vehicles—Anthropomorphic side impact dummy—Lateral impact response requirements to assess the biofidelity of the dummy. 1999.

Kleiven, S., 2007. Predictors for traumatic brain injuries evaluated through accident reconstructions. Stapp Car Crash J 51, 81-114.

Post, A., Koncan, D., Kendall, M., Cournoyer, J., Michio Clark, J., Kosziwka, G., Chen, W., de Grau Amezcua, S., Blaine Hoshizaki, T., 2018. Analysis of speed accuracy using video analysis software. Sports Engineering 21, 235-241.

**Supplementary Table 1**

| Tissue | Density [kg/m3] | 𝜇1 [Pa] | 𝛼1 | 𝜇2 [Pa] | 𝛼2 | Bulk modulus [MPa] |
| --- | --- | --- | --- | --- | --- | --- |
| Brain | 1040 | 53.8 | 10.1 | -120.4 | -12.9 | 50 |
| Brain stem | 1040 | 15.8 | 28.1 | -106.8 | -29.5 | 50 |
| 𝜏𝑖 [ms] | 𝜏1 = 0.001 | 𝜏2 = 0.01 | 𝜏3 = 0.1 | 𝜏4 = 1 | 𝜏5 = 10 | 𝜏6 = 100 |
| 𝐺𝑖 [kPa] | 𝐺1 = 320 | 𝐺2 = 78 | 𝐺3 = 6.2 | 𝐺4 = 8.0 | 𝐺5 = 0.1 | 𝐺6 = 3.0 |

**Supplementary Table 2**

| Tissue | Density [kg/m3] | 𝜇1 [kPa] | 𝛼1 | Poisson’s ratio |
| --- | --- | --- | --- | --- |
| SAS and ventricles | 1040 | 20 | 2 | 0.4998 |
| Falx and tentorium | 1130 | 25.5 | 32.9 | 0.45 |
|  | 𝜏𝑖 [ms] | 𝜏1 = 5 | 𝜏2 = 44 | 𝜏3 = 474 |
|  | 𝐺𝑖 [kPa] | 𝐺1 = 328 | 𝐺2 = 291 | 𝐺3 = 161 |
| Pia mater | 1130 | 2.6 | 32.9 | 0.45 |
|  | 𝜏𝑖 [ms] | 𝜏1 = 5 | 𝜏2 = 44 | 𝜏3 = 474 |
|  | 𝐺𝑖 [kPa] | 𝐺1 = 32 | 𝐺2 = 29 | 𝐺3 = 16 |
